# SFPQ and Its Isoform as Potential Biomarker for Non-Small-Cell Lung Cancer

**DOI:** 10.3390/ijms241512500

**Published:** 2023-08-06

**Authors:** Libang Yang, Adam Gilbertsen, Blake Jacobson, Jenny Pham, Naomi Fujioka, Craig A. Henke, Robert A. Kratzke

**Affiliations:** 1 Department of Medicine, University of Minnesota, 420 Delaware Street, SE, Minneapolis, MN 55455, USA; yangx822@umn.edu (L.Y.); gilbe398@umn.edu (A.G.); henke002@umn.edu (C.A.H.); 2 Hematology, Oncology and Transplantation, School of Medicine, University of Minnesota, 420 Delaware Street, SE, Minneapolis, MN 55455, USA; jacob023@umn.edu (B.J.); fujio002@umn.edu (N.F.); 3 Clinical and Translational Science Institute, School of Medicine, University of Minnesota, 420 Delaware Street, SE, Minneapolis, MN 55455, USA; pham0435@umn.edu

**Keywords:** SFPQ, cytoplasmic isoform, metastasis, non-small cell lung cancer (NSCLC), ELISA, IHC, DNA methylation

## Abstract

Cancer markers are measurable molecules in the blood or tissue that are produced by tumor cells or immune cells in response to cancer progression. They play an important role in clinical diagnosis, prognosis, and anti-drug monitoring. Although DNA, RNA, and even physical images have been used, proteins continue to be the most common marker. There are currently no specific markers for lung cancer. Metastatic lung cancer, particularly non-small-cell lung cancer (NSCLC), is one of the most common causes of death. SFPQ, YY1, RTN4, RICTOR, LARP6, and HELLS are expressed at higher levels in cells from NSCLC than in control or cells from inflammatory diseases. SFPQ shows the most difference between the three cell types. Furthermore, the cytoplasmic isoform of SFPQ is only found in advanced cancers. We have developed ELISAs to detect SFPQ and the long and short isoforms. Evidence has shown that the short isoform exists primarily in cancers. Furthermore, immunocytometry studies and IHC analysis have revealed that SFPQ levels are consistent with ELISA results. In addition, enhanced DNA methylation in the SFPQ gene may facilitate the SFPQ expression differences between control and cancer cells. Considering this, elevated SFPQ level and the isoform location could serve as a cancer diagnostic and prognostic marker.

## 1. Introduction

Cancer markers are measurable molecules that circulate in the blood or tissue that are generated by tumor cells or immune cells and correspond with cancer progression. Cancer markers are crucial in clinical diagnosis, prognosis, and cancer therapy monitoring. Non-small cell lung cancer (NSCLC) is one of the most common cancers that often leads to death. Several proteins, including CK20, CDX2, STAT3, and CYFRA21-1, have been found to contribute to tumor growth and metastasis, and could potentially serve as diagnostic markers for distant metastasis [1,2,3]. However, no specific markers for lung cancer, as well as many other cancers, are currently known [4,5,6,7]. Discovery of the key genes or proteins that contribute to lung cancer development and progression could greatly help in understanding the development of lung cancer and the discovery of novel therapeutic targets. These could serve as markers and play a pivotal role in clinical diagnosis, prognosis, and choice of therapeutic agent.

Our proteomics analysis discovered many protein expression differences between NSCLC and controls. A panel of these differentially expressed proteins was analyzed further to determine if they are suitable to serve as biomarkers. YY1, RTN4, RICTOR, SFPQ, LARP6, and HELLS have been associated with cancers previously [8,9,10,11,12,13,14] and, in this study, exhibited differential level changes between control, Idiopathic Pulmonary Fibrosis (IPF) and lung cancer cells. IPF is a progressive and malignant disease that is different from lung cancer because it does not metastasize. SFPQ is not only high in NSCLC, but its cytoplasmic isoforms are also present only in advanced stages of lung cancer. In light of this, we have developed sandwich ELISAs to detect the SFPQ isoforms that exist in cell lysates, cell medium, and human serum. When employing these novel ELISAs, it was discovered that SFPQ levels are high in lung cancer and other cancer samples. Additionally, both IHC and IF also found that in the cytoplasm, SFPQ isoform is elevated in cancer cells. Considering this, SFPQ expression and its subcellular location is a special feature and can serve as a diagnostic and treatment-monitoring marker in lung cancer.

## 2. Result

### 2.1. SFPQ Has Highest Expression in a Panel of Overexpressed Proteins in Lung Cancer Compared to IPF and Other Cellular Controls

In previous studies, we used the cell surface markers CD44 and stage-specific embryonic antigen-4 (SSEA-4) to isolate stem-like cells from primary fibroblasts derived from explant IPF tissue. It has been shown that CD44+SSEA-4+ double-positive cells preferentially express some stem-cell genes [15,16]. CD44 and SSEA-4 were used as markers for the isolation of stem cells from the NSCLC and normal lung cells [17]. We found several proteins differentially expressed between the control and NSCLC. To screen NSCLC markers further, we include IPF nuclear protein profiles in our comparison. IPF is a progressive and malignant disease that is different from lung cancer because it does not metastasize. Three groups of nuclear proteins (ProteomeXchange: PXD032352) were applied further to ingenuity signal transduction pathway analysis (www.ingenuity.com, accessed on 1 February 2023), then the selected proteins were further screened with the Proteinatlas database (www.proteinatlas.org, accessed on 1 February 2023).

Global proteomic analyses with stem cells from NSCLC, IPF, and the control group identified 5016 proteins (4189 unique entries) present in the nuclear fraction of all cell groups. A total of 1041 proteins (20% of the total protein) were observed to be significantly different (with *p* < 0.05 in the Mann–Whitney Test) between the control and IPF groups, 981 proteins (20% of the total protein) were observed to be significantly different (with *p* < 0.05 in the Mann–Whitney Test) between the control and NSCLC groups; 469 proteins were observed to be significantly different between NSCLC and IPF groups. Those proteins were applied to Ingenuity Pathway Analysis (IPA) software to analyze biomarkers, cell function, upstream regulatory factors, and signal transduction pathways. YY1, RTN4, RICTOR, SFPQ, BMI1, LARP6, and HELLS expression levels were differentially elevated in NSCLC compared to IPF and controls, while this panel of proteins had the greatest expression in NSCLC. When we review the details of those proteins, they are all related to cancer development [8,9,10,11,12,13,14]. SFPQ [18,19,20], RTN4 [12,21], RICTOR [22,23,24,25,26], LARP6 [27,28,29,30], and HELLS [8,9,10,31,32] are all associated with cancer.

**Figure 1:**
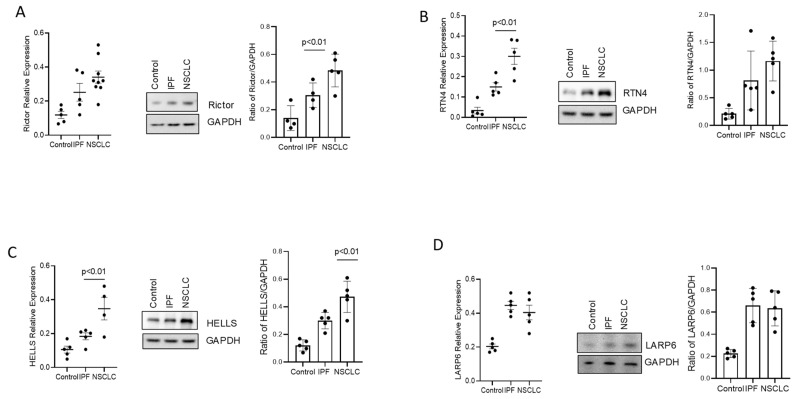
Differential expression levels of RICTOR, RTN4, HELLS, and LARP6 among NSCLC, IPF, and controls. Primary cells from controls, IPF, and NSCLC were used in RT-PCR analysis and Western blot to quantify RICTOR (**A**), RNT4 (**B**), HELLS (**C**), and LARP6 (**D**). Significance was analyzed using One-Way ANOVA.

**Figure 2:**
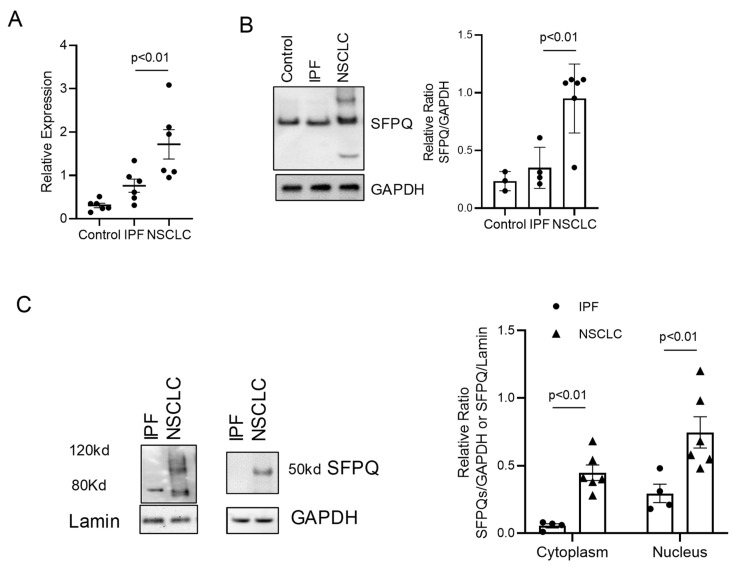
The expressions of SFPQ isoforms are different between NSCLC, IPF, and controls. SFPQ levels were analyzed with RT-PCR (**A**) and Western blot analysis (**B**). Densitometry values are shown in the right-hand graph. Multiple SFPQ bands present in NSCLC, IPF, and controls are the different SFPQ isoforms. SFPQ expression levels are different between IPF and NSCLC in Western blot analysis with primary cell lysates. (**C**) The different SFPQ isoforms in cytoplasmic and nuclear fractions from NSCLC, IPF, and control primary cell lines. The left panel shows an example of Western blot bands for SFPQ in the nuclear fraction of those cells (Lamin as loading marker). The right panel shows an example of Western blot bands for SFPQ in the cytoplasm fraction of those cells (GAPDH as a loading marker). Significance was analyzed using One-Way ANOVA.

Each protein involved in cell processing was probed in the Proteinatlas database (https://www.proteinatlas.org/, accessed on 1 June 2023) and investigated for the largest expression difference in NSCLC cells. SFPQ, RTN4, HELLS, RICTOR, and LARP6 have high expression in lung cancer and other cancer cells and tissues. We further conducted RT-PCR and Western blot analysis to validate the expression level of RTN4, RICTOR, LARP6, and HELLS with primary control cells compared to IPF and NSCLC cells. RTN4, RICTOR, and HELLS have elevated expressions in NSCLC cells compared to normal control and IPF cells. LARP6 was shown to be higher in IPF than in NSCLC or normal controls. We further analyzed these proteins using Western blot studies and found RICTOR and LARP6 have greater expressions in both IPF and NSCLC but have similar expressions for NSCLC and IPF (Figure 1A,D). SFPQ, RTN4, and HELLS were shown to have high expression in both IPF and NSCLC compared to control cells, and a substantial difference between IPF and NSCLC was revealed (Figure 1B,C, and Figure 2A,B). The protein with the largest expression difference in NSCLC compared to control cells is SFPQ.

### 2.2. SFPQ Isoform Levels Are Different between Control and Lung Cancer Cells

Splicing factor proline- and glutamine-rich (SFPQ) is a ubiquitous and abundant RNA binding protein (RBP) that plays multiple regulatory roles in the nucleus such as paraspeckle formation, DNA damage repair, and several transcriptional regulation processes. An increasing number of studies have demonstrated the nuclear and cytoplasmic roles of SFPQ in neurons, particularly in post-transcriptional regulation and RNA granule formation. Not surprisingly, the misregulation of SFPQ has been linked to pathological features shown by other neurodegenerative disease-associated RBPs such as aberrant RNA splicing, cytoplasmic mislocalization, and aggregation. SFPQ has been implicated in numerous cancers often due to interactions with coding and non-coding RNAs, along with some nuclear proteins [18,19,20]. The SFPQ cytoplasm isoform was reported to be related to neuronal diseases [33]. To determine if there is an SFPQ expression-level difference extensive enough between normal and NSCLC for SFPQ to serve as a diagnostic marker, RT-PCR and Western analysis were performed. SFPQ was shown to be higher in lung cancer than control and IPF cells, or inflammatory lung disease cells (Figure 2A). Subcellular fractions of controls, IPF, inflammatory lung disease cells, and lung cancer cells were isolated and used to analyze SFPQ isoforms. Antibodies against the C-terminal of SFPQ and a segment near the N-terminal of SFPQ were used in Western analysis. Bands of 80 and 120 kDa SFPQ were detected in most of the cellular nuclear fractions while the 30 and 50 kDa SFPQ bands were observed in NSCLC cells (Figure 2B,C). SFPQ levels were 5-fold higher in NSCLC than in control and 2.5-fold higher than IPF revealed by RT-PCR. SFPQ exhibited 4-fold higher levels than control samples and 2-fold higher expression than IPF in Western blot analysis (Figure 2). These data indicate that SFPQ is elevated in lung cancer, and the short SFPQ is exclusively elevated in the cytoplasm of lung cancer cells. Given this, the short SFPQ isoform may be considered a specific marker for NSCLC.

### 2.3. Short SFPQ Isoform Is Only Located in the Cytoplasm of Lung Cancer Cells

**Figure 3:**
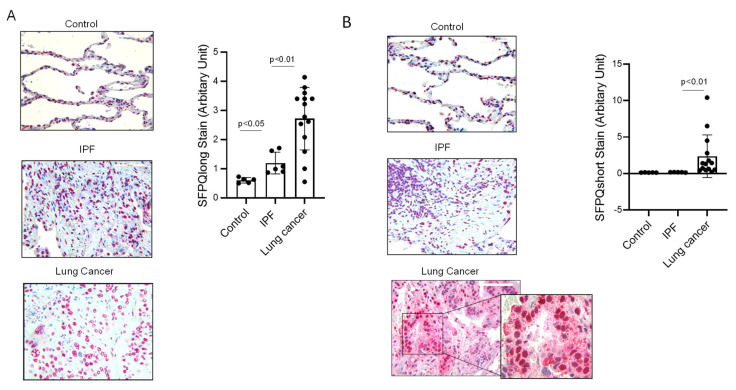

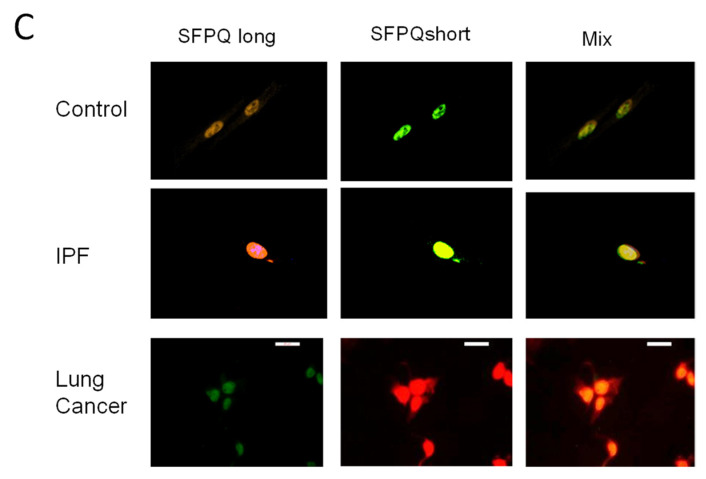
SFPQ short isoform exists in NSCLC cytoplasm, but SFPQ long isoform is in the nucleus of all tested cells. (**A**) IHC staining of lung cancer tissue, IPF, and control. SFPQ antibodies were used to detect SFPQ in the samples. A. SFPQ antibody HPA054689 (polyclonal, antigen: C-terminal peptide) for full-length SFPQ and (**B**) SFPQ antibody CAB009886 (Polyclonal, antigen: whole SFPQ) for short and long SFPQ. (**C**) Immunostaining lung cancer tissue, IPF, and control for SFPQ isoforms. An antibody to SFPQ and an antibody to SFPQ C-term (Anti-C-terminal antibody) were employed to detect the long and whole SFPQ isoforms. In addition, an antibody to the SFPQ N-terminal was used to recognize the short and long isoforms. Significance was analyzed using one-way ANOVA.

Since the short isoform of SFPQ is in the cellular fraction of lung cancer as shown in the immunoblot analysis employing cell lines, we further examined if this phenomenon persists in lung cancer from tissue samples. IHC and IF with anti-short or long SFPQ were developed and applied to cell lines and patient samples. Anti-SFPQ short (against N-terminal peptide) or SFPQ long (against C-terminal peptide) were used in IHC and IF staining. The results from IHC showed that SFPQ long exists in the nucleus of normal control, IPF, and lung cancer, while lung cancer had the highest expression level (Con: IPF: Cancer = 0.6044 + 0.0175:1.17 + 0.024:2.76 + 0.21) (Figure 3A). We tested 14 cases of lung cancer; 11 lung cancers were positive for the anti-N-terminal of the SFPQ antibody (short SFPQ) in the cytoplasm of cancer cells. Alternatively, in 9 non-cancer tissues, the cytoplasm was negative for SFPQ N-terminal antibody (short SFPQ) (Con: IPF: Cancer = 0.134 + 0.0115:0.17 + 0.024:2.56 + 0.23) (Figure 3B), indicating SFPQ isoforms are located differentially into subcellular compartments. IF staining with the primary cell lines led to a similar result; however, the results displayed a positive anti-SFPQ N-terminal stain with lung cancer cells but not control and IPF cells (Figure 3C). Since the short SFPQ isoform is only present in the cytoplasm of lung cancer cells, this SFPQ isoform distribution could be a potential marker for clinical tests for lung cancer.

### 2.4. ELISA Assay for SFPQ Isoforms

**Figure 4:**
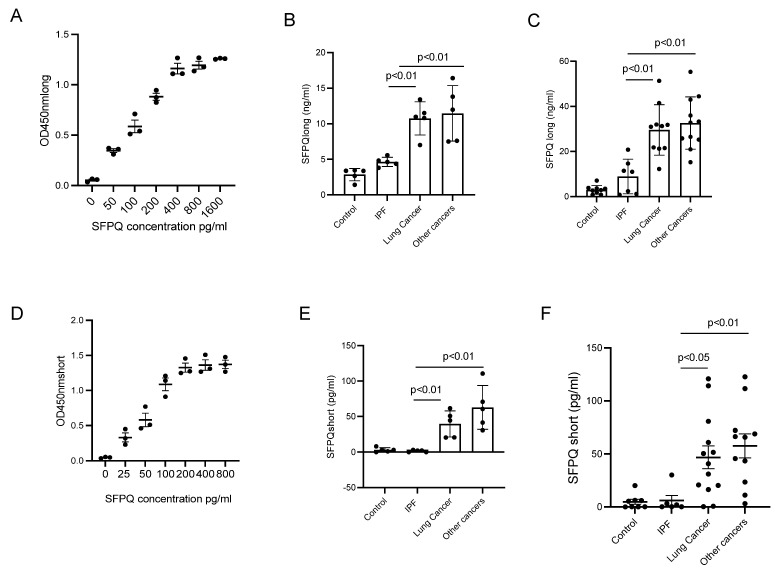
SFPQ isoform levels are high in NSCLC cell medium and NSCLC patient serum. (**A**) SFPQ long ELISA can detect SFPQ in samples from 20 pg or greater. (**B**) Measurement of SFPQ long in control, IPF, and cancer cell medium with ELISA. (**C**) SFPQ long is measured in control, IPF, and cancer patient serum. (**D**) SFPQ short ELISA can detect SFPQ lower than 10 pg per sample. (**E**) SFPQ short is measured in control, IPF, and cancer cell medium with ELISA. (**F**) SFPQ short is measured in control, IPF, and cancer patient serum. Cell lines for (**B**,**E**): 5 lung cancer; another cancer: 3 breast cancer, 2 kidney cancer. Serum for (**C**,**F**): 13 lung cancer, 4 kidney cancer, 4 breast cancer, and 3 prostate cancer. Significance was analyzed using one-way ANOVA.

To determine if SFPQ could be a diagnostic marker, it was necessary to ascertain if SFPQ is detectable in human serum. ELISAs for both SFPQ isoforms were developed to generate a differential ELISA assay system to assess both isoforms. SFPQ levels in cancer cell lines and lung cancer patient serum were greater than IPF and control cell lines and patient serum. SFPQ was detected with SFPQ long ELISA in all cell lysates and cell-conditioned medium. The assay could detect SFPQ as low as 20 pg/mL (Figure 4A). SFPQ long in all cancer cell-conditioned medium (Figure 4B) were much higher than control and IPF cell conditional medium. To measure SFPQ short isoform, the SFPQ long isoform was depleted from samples using SFPQ long antibody affinity column; then, the samples were applied to SFPQ short ELISA. SFPQ short ELISA could detect 5~160 pg/mL of SFPQ short (Figure 4D). SFPQ short was only detected in cell conditioned medium from cancer cells (Figure 4E).

### 2.5. SFPQ Is Elevated in Cancer Serum

We further measured the SFPQ level using ELISAs with human serum samples from control disease patients, IPF patients, and cancer patients. For SFPQ long, samples were diluted 20-fold and then applied to SFPQ long ELISA. For the SFPQ short, samples were diluted 2-fold and then applied to an SFPQ long isoform affinity column followed by ELISA. SFPQ was shown to be higher in cancers than in controls and IPF. The SFPQ long isoform was shown to have higher levels in cancer than in other diseases, while SFPQ short isoform was mainly detected in cancer samples (Figure 4C,F). The long SFPQ isoform detected by ELISA in control and IPF (3.34 + 0.24 and 9.97 + 1.24 ng/mL) was shown to be much lower than in lung cancer and other cancer serums (40.94 + 2.81 and 43.57 + 2.64 ng/mL). Short SFPQ isoform levels in cancer serum were 46.84 + 3.55 in lung cancer and 55.17 + 4.34 pg/mL in serum from other cancers (compared to 4.76 + 0.82 in control and 6.64 + 1.95 pg/mL in IPF). These data suggest that SFPQ long levels were higher in lung cancer patient serum and serum from other cancer patients than in serum from other diseases, whereas SFPQ short levels were found primarily in lung cancer patient serum and serum from other cancer patients. The short SFPQ level change is more cancer-specific.

### 2.6. DNA Methylation Affects SFPQ Expression in Lung Cancer and Other Cancers

We then explored a possible mechanism for the SFPQ isoform changes seen in lung cancer. DNA methylation is an important factor in cancer development [34,35,36,37,38,39] that can lead to changes in expression [40,41,42]. We have discovered DNA methylation differences in the SFPQ promoter region which may be responsible for the SFPQ isoform expression differences between lung cancer and IPF and normal cells.

**Figure 5:**
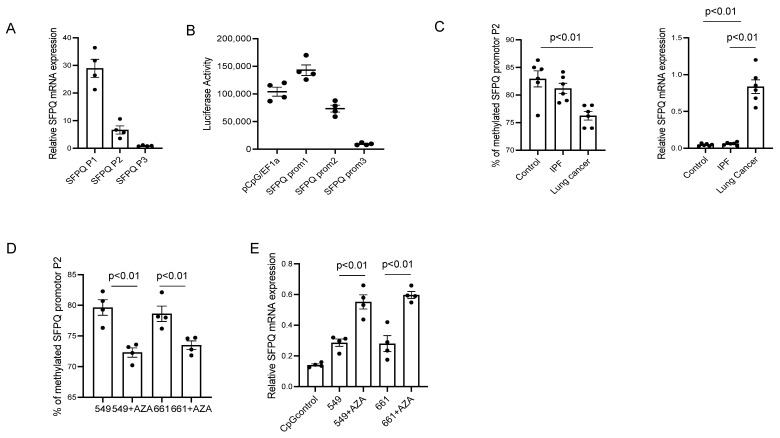
The methylation levels of SFPQ promoters are inversely related to SFPQ expression. (**A**) RT-PCR was employed to quantify SFPQ isoform mRNA levels. (**B**) To characterize the SFPQ promoter segments, several luciferase reporter constructs were generated to determine promoter strength. *n* = 4 each in figure (**A**–**C**) Methylation levels of SFPQ Promoter P2 (275 bp) in primary cell lines of normal, IPF, and lung cancer were assessed, (*n* = 8 each). (**D**) The quantification of SFPQ P2 isoform levels measured by RT-PCR. (**E**) The methylation in SFPQ promotor P2 affects SFPQ expression. Methylation levels of SFPQ promote P2 (bp 275) in primary cell lines of normal and NSCLC were determined by the sequencing of clones subjected to bisulfite conversion [left graph]. The right graph reveals SFPQ isoform levels quantified by RT-PCR—a total of 4 cell lines with 3 separate experiments. Significance was analyzed using one-way ANOVA.

The different-sized SFPQ isoforms could be due to protein expression from multiple promotors in the regulatory region of its gene. Given this, levels of the various-sized SFPQ mRNA were assessed. These studies revealed that segment P1 is substantially elevated compared to segment P2 which has a greater level than segment P3 (Figure 5A). These results suggest that there are different isoforms expressed at different levels. The SFPQ upstream region was analyzed with a promoter program [43], and it was revealed that there are three promotors in this region. The promotor activity was determined after inserting these three different DNA segments into a luciferase reporter vector [44] (Figure 5B). The result demonstrates that Promoters 1 and 2 have higher activity than promotor 3. Promotor 2 should be responsible for the expression of short SFPQ based on promotor activity level and SFPQ isoform expression level. To test if the DNA methylation status induces the SFPQ isoform level variation in lung cell lines, the DNA methylation levels were assessed in the Promoter 2 region in lung cancer and control cells. It was revealed that the DNA is hypomethylated in the SFPQ promoter of cancer cells compared with control cells and IPF cells (Figure 5C). In addition, the mRNA level of the P2 segment of SFPQ is different between control, IPF, and lung cancer cells (Figure 5D). mRNA levels were very low in the control and IPF samples, which is consistent with Western blot analysis earlier. Then, we examined if methylation inhibition changed the methylation level and SFPQ expression. Following the inhibition of DNA methylation in lung cancer cells, the DNA methylation level in SFPQ promoters decreased, which led to an increase in SFPQ expression (Figure 5E). These data indicate that DNA methylation causes abnormal SFPQ isoform expression.

## 3. Discussion

Cancer markers are measurable molecules that circulate in the blood or tissue and are generated by tumor cells or immune cells in response to cancer progression (3, 4, 21, 22). They play an important role in clinical diagnosis, prognosis, and anti-drug monitoring. Although DNA, RNA, and even physical images have been used, proteins remain the most common marker type. Cancer markers have several advantages over traditional diagnostic approaches, including being less expensive, less time-consuming, and less invasive. NSCLC is one of the most common cancers that kills. Numerous clinical trials have been conducted to look for markers for advanced-stage NSCLC. Some markers, including CEA, CA19-9, CA125, AFP, NSE, CK20, CDX2, STAT3, CA15-3, and CYFRA21-1, were investigated, but none were found to be specific to cancer [45,46]. MSCs have recently received a lot of attention for their role in the development of various diseases and cancers. They may aid in tumor growth and metastasis [45,46,47,48,49]. Spatial proteomics is a powerful, evolving technology that defines the proteome in specific subcellular compartments [50,51]. The combination of quantitative mass spectrometry and bioinformatics analysis has a significant advantage in screening key proteins for specific cell functions [52,53,54,55,56,57,58]. We used quantitative mass spectrometry and creative pathway analysis to identify markers in the nuclear compartment of NSCLC and IPF MSCs, which has a few advantages. (1) Using proteins from a nuclear fraction (rather than proteins from the whole cell) avoids common protein noise in proteomics analysis; (2) Ingenuity pathway analysis excludes proteins from common structures, pathways, and functions; and (3) Data showed that several proteins were identified with significantly different levels in NSCLC and controls. They include YY1, RTN4, RICTOR, SFPQ, BMI1, and HELLS. They are not only higher in NSCLC but also high in other cancers. SFPQ was not only much higher in NSCLC tissue but also a 50 KD isoform of it was mostly found in cancer samples.

SFPQ is a multifunctional protein. It can bind to DNA and RNA and thus regulate RNA splicing and protein transcription. It was also discovered that its accumulation in the cytoplasm could be the cause of some neuronal diseases [20,21,34,47]. SFPQ levels are elevated not only in lung cancer but also in other cancer samples. SFPQ mRNA and protein levels were first assessed in control and lung cancer primary cells; SFPQ levels are higher in lung cancer cells than in other cells. When we measured SFPQ in patient serum, we discovered that it is higher in cancer serum than in other serum samples. Because SFPQ serves multiple functions in cells, changes in its expression level may have an impact on cell function and its cytoplasmic isoform. First, we found its cytoplasm isoform is exclusively present in advanced-stage NSCLC. Then we discovered this isoform also exists in other lung cancer and other solid cancers. It is also found in the serum of cancer patients. As a result, SFPQ and its cytoplasm (short) isoform are appropriate diagnostic markers for these cancers. The difference was detected by ELISA, and IHC and immunocytochemistry revealed that SFPQ isoforms are present in different subcellular locations. Then, all ELISA, IHC, and immunocytostaining techniques could be used to detect abnormalities in SFPQ levels and subcellular location. Serum and bodily fluids are the best clinical samples for disease diagnosis. We used an ELISA to detect SFPQ in cell lysates, cell conditional medium, and patient serum. The current findings are based on a small number of patients with advanced cancers; however, with more human patient samples, the relationship between SFPQ levels and SFPQ isoform subcellular levels and cancer stages could be established.

DNA methylation is essential for human life, and it is an epigenetic phenomenon of adding a methyl group to the 5 position of the cytosine without changing DNA sequences. This epigenetic modification can alter gene expressions and could be inherited by the next generation [47,48]. The DNA methylation process plays an important role in the life of mammals because this process regulates aging and cancer [49,50]. It is well known that aberrant DNA methylation is one of the chief factors associated with many forms of cancers, and usually, many cancer-related genes contain hypermethylation. In addition, promoter gene methylation is better understood than methylation in non-promoter regions, and whether gene methylation in the non-promoter region has the same significance for cancers is largely unclear. Promoter DNA methylation can prevent tumor suppressor gene expression. Most CGIs (CpG islands) are subjected to methylation [38,39]. In summary, aberrant changes in DNA methylation could facilitate tumor development. Our findings revealed that there are three promoter regions in SFPQ upstream, with varying levels of methylation. SFPQ levels in cancer cells were altered by manipulating their methylation. These findings suggest that DNA methylation in SFPQ promotor regions may regulate SFPQ expression differently in cancers compared to normal control and inflammatory lung diseases. The effect of SFPQ on cancer cell function is not the focus of this paper. Because SFPQ has multiple functions in cells, changes in its level should affect cell functions in cancer cells. The cytoplasm isoform is another potential mechanism for SFPQ in cancer pathology. There are few reports on its mechanism in neuronal diseases, and cytoplasm SFPQ plays a key role in the development of those neuronal diseases [33,51,52,53]. SFPQ could bind RNA, and RNA binding of its abnormal cytoplasm isoform might play roles in cancer cells too.

Our findings suggest that SFPQ and its isoforms may serve as markers for lung cancer and other solid cancers. More research will be needed to determine whether it holds with a larger number of patient cases and samples.

## 4. Materials and Methods

### 4.1. Primary Cell Lines, Patient Tissue Sections and Serum

Primary lung cell lines were established from patients fulfilling diagnostic criteria for IPF and lung cancer including a pathological diagnosis of usual interstitial pneumonia. Patient controls were selected to be similar in age to IPF and lung cancer patients with non-fibrotic lung disorders. Control, IPF, and lung cancer cell lines were derived from lungs, and cultivated as previously described [54]. Human serum and patient tissue sections were collected and prepared through Bionet, University of Minnesota. Serum was collected and stored at −80 °C for further analysis.

### 4.2. Cell Cultures and FACS Sorting

Primary cells for NSCLC and control cases were harvested from the lung tissue biopsy of adult donors according to a protocol approved by the University of Minnesota Institutional Review Board. Culture supplies were obtained from Thermal Scientific except where noted. MSCs were enriched, purified, and cultured as described previously [16,55,56]. For the isolation of MSCs, primary mesenchymal cells were labeled with mouse anti-human SSEA4 antibody conjugated to Alexa Fluor^®^ 647 (Clone MC-813-70; Catalog #560796; BD Biosciences, Franklin Lake, NJ, USA) and mouse anti-human CD44 conjugated to FITC (Clone IM7; Catalog #103006; BioLegend, San Diego, CA, USA). Cells were sorted on a FACS Aria Cell Sorter (BD Biosciences). Cells with SSEA4+ and CD44+ (relative to mouse IgG3 κ isotype control conjugated to Alexa Fluor^®^ 647, clone J606, catalog #560803 BD Biosciences and mouse IgM κ isotype control conjugated to FITC, catalog #402207; BioLegend, respectively) were collected as previously described [55]. For IPF MSC isolation, the FACS Sorter gate was set to collect SSEA4-positive cells at the top 3% of CD44 expression.

### 4.3. Isolation of Cell Nucleus

Primary MSCs were used to isolate cell nuclei with the cell organelles fraction kit (Thermo Scientific, Waltham, MA, USA) by following the manufacturer’s instructions. Nuclear fractions of lung cancer MSCs and control MSCs were isolated by NE-PER Nuclear and Cytoplasmic Extraction reagents (Thermo Scientific, Waltham, MA, USA).

### 4.4. Ingenuity Pathway Analysis (IPA)

The lung cancer MSC nuclear proteomic analysis data were imported to the IPA (http://www.ingenuity.com, accessed on 1 March 2023) for functional analysis, canonical pathways, and upstream regulator analysis. Fisher’s exact test was used to calculate a *p*-value, which determines the probability that each biological function and/or disease assigned to the dataset is caused only by chance [57,58].

### 4.5. Assay for SFPQ Isoforms

SFPQ ELISAs were established. SFPQlong ELISA: Antibody for full-length SFPQ was used for coating (2 μg/mL each, 15585-1, Proteintech); detecting antibody was H00006421, Abnova for full-length SFPQ and HRP-anti-mouse or rabbit conjugate. SFPQshort ELISA employed an antibody against SFPQ amino acids from 236 to 349 was used for coating (2 μg/mL each, 15585-1, Proteintech). Detecting antibodies were 67129-1, Proteintech for short isoform, and HRP-anti-mouse or rabbit conjugate.

Serum, plasma, tissue homogenates, or cell lysate and cell culture medium could be applied. For long SFPQ, all samples were diluted and applied directly. For SFPQ short, all samples were gone through the SFPQ C-terminal column first and then applied to ELISA. SFPQ C-terminal Ab affinity column was used to deplete full long SFPQ in samples. A total of 10 μg of anti-SFPQ C-terminal antibody (122-133112, Raybiotech Peachtree Corners, GA, USA) was cross-linked with Mag Sepharose beads (2894409, Cytiva Waltham, MA, USA) to make this affinity column.

### 4.6. IHC (Immunohistochemistry) Staining for SFPQ

Immunohistochemistry was performed on 4 µm paraffin-embedded serial sectioned NSCLC and control lung tissue and mounted on polylysine-coated slides. The sections were deparaffinized in xylene, rehydrated through a graded methanol series, quenched with 0.3% hydrogen peroxide in methanol, and immersed in a 98 °C water bath for 30 min in citrate buffer (pH 6.0) for antigen retrieval. Sections were placed in 5% Normal Horse Serum (Jackson Immunoresearch, West Grove, PA, USA) to block the non-specific binding of secondary antibodies. A multiplex immunohistochemistry kit was used for antigen detection according to the manufacturer’s instructions (MULTIVIEW IHC Kit ADI-950-101-0001; Enzo Life Sciences, Farmingdale, NY, USA). The tissue specimens were incubated overnight (18–20 h, 4 °C) with the following primary antibodies: anti-SFPQ monoclonal antibody (1:800) (Ab1888647, Abcam Waltham, MA, USA), anti-human SFPQ antibody (1:500) (Ab15086, Abcam, USA). Specimens were cover-slipped with a Prolong Antifade Kit (Invitrogen/Molecular Probes) and stored overnight at room temperature without light before image analysis. The tissue section was then visualized with DAB or Vulcan Fast Red (Biocare Medical Pacheco, CA, USA).

### 4.7. DNA Methylation Analysis

Coupling bisulfite conversion with DNA sequence analysis was used [59]. EZ DNA methylation direct kit was used (Zymoresearch, Irvine, CA, USA). The methylation levels of CpG sites were evaluated with pyrosequencing. All manipulations of bisulfite conversions, PCRs, and methylation quantification were previously described [60].

### 4.8. Luciferase Reporter Assay

Promoter segments (P1, bp 214~516; P2, bp 800~1075; P3, 9413~9685) of SFPQ were inserted into the luciferase vector and luciferase activity was then measured [44] (Renogen, Vancouver, Canada). A Luciferase reporter gene detection kit was used. The assay was conducted following the manufacturer’s instructions (Millipore Burlington, MA, USA). To perform the luciferase assay, 100 μL of each plate lysate is used for luciferase assay. Results were read at 480 nm with a SpectraMax M3 microplate reader (Molecular Devices San Jose, CA, USA).

### 4.9. Western Blot Analysis

Cells were washed twice in cold PBS and lysed in New RIPA lysis buffer (150 mM NaCl, 50 mMTris pH 8.0, 1 mM EDTA, 1 mM EGTA, 0.5% sodium deoxycholate, 0.1% SDS, and 1% Triton X-100) with protease inhibitor cocktail (0.1 M phenylmethylsulfonyl fluoride, 5 μg/mL leupeptin, 2 μg/mL aprotinin, and 1 μg/mL pepstatin). Protein concentrations of whole cell lysates were determined using the BCA method and equal amounts of each protein sample (15 μg) were separated on an 8~14% SDS–polyacrylamide gel at 80 V. Separated proteins were then transferred to a polyvinylidene difluoride membrane for 8 min on the turbo transfer system (Invitrogen Carlsbad, CA, USA). After blocking with 5% skim milk powder for 1 h at RT, the membrane was incubated with primary antibody for 1 h at RT or overnight at 4 °C. The membrane was washed three times for 15 min with 0.05% PBS-Tween and then incubated for 1 h at RT with the horseradish peroxidase-conjugated secondary antibody. After extensive washing with 0.05% PBS-T, protein bands were visualized by ECL Plus according to the manufacturer’s instructions (Cell signaling Danvers, MA, USA).

### 4.10. Real-Time Reverse Transcription PCR

Total RNA was extracted with the RNeasy minikit and the cDNA was synthesized with miScript 92 RT kit (Qiagen Hilden, Germany). PCR reactions contained 10 μL SYBR@Green SuperMix (Bio-Rad Hercules, CA USA), 900 nM forward primer, 900 nM reverse primer, and 50 ng cDNA in 20 μL of reaction volume. Reactions were performed in a 7900 HT Sequence Detector (Applied Biosystems Waltham, MA USA) with a cycling protocol (conditions: pre-denaturation at 95 °C for 5 min. For the cyclic reactions (40×); denaturation at 95 °C for 5 s and amplification at 60 °C for 30 s. The fluorescence reading was performed at the amplification step) described before (Applied Biosystems) [55]. The primers are as follows:
GAPDH Forward: 5′-TGTTGCCATCAATGACCCCTT-3′
GAPDH Reverse: 5′-CTCCACGACGTACTCAGCG-3′
Rictor Forward: 5′-TGTATGCAAGAGCCAAGCAC-3′
Rictor Reverse: 5′-CTGATTCCTGCTTTCCACAAG-3′
RTN4 Forward: 5′- GGCTCAGTGGATGAGACCCT-3′
RTN4 Reverse: 5′- TGTTACCTGGCTGCTCCTTC-3′
HELLS Forward: 5′-TAGAGAGTCGACAGAAATTCGG-3′
HELLS Reverse: 5′-CCTCATAACTGGCTTCTCTTCA-3′
LARP6 Forward: 5′- TTACACGGGACTGGAGAACC-3′
LARP6 Reverse: 5′- GTCCCAAAAAGCTTGAGCAG-3′
SFPQ Forward: 5′-GATCTACAGGGAAAGGCATTGTTG-3′
SFPQ Reverse: 5′-GATACATTGGATTCTTCTGGGCA-3′
SFPQ isoform PCR primers:
SFPQ P1 Forward: 5′-CTCCACGACTTCCGTTCTCC-3′
Forward: 5′-CTGAGGAGGTGTGGTAGGGA-3′
SFPQ P2 Forward: 5′-GTCCCTACCACACCTCCTCA-3′
Forward: 5′-TCCGAGATCTTCTCCTCGCT-3′
SFPQ P3 Forward: 5′-GCAGCGAGGAGAAGATCTCG-3′
Forward: 5′-CTGTCGTCTCATCAGATAGGTCTT-3′
SFPQ Promotor1
Forward: 5′-GCCTCAATCAGAATCGCGG-3′
Reverse: 5′-GGTCGGAGTCGGGCCT-3′GGTCGGAGTCGGGCCTSFPQ Promotor 2
Forward: 5′-GGTCCCAAAGGCGGCAAAAT-3′
Reverse: 5′-CCATCTTAGGGGAGCCGAC-3′
SFPQ Promotor 3
Forward: 5′-GGTCATTTTGTTGCATTTCCCC-3′
Reverse: 5′-TTTGCCCAACAGAAGTAGCAC-3′


RT-PCR products were quantified at the log-linear portion of the curve using LightCycler analysis software and compared to an external calibration standard curve.

### 4.11. Statistical Analysis

All experiments were performed at least in triplicate, and results were analyzed using one-way ANOVA (For the proteomics method described above). The criterion for significance was *p* < 0.05. Numerical data are reported as means ± standard deviations.

## Data Availability

ProteomeXchange: PXD032352.

## References

[1] Jin X., Zhu Z., Shi Y. (2014). Metastasis mechanism and gene/protein expression in gastric cancer with distant organs metastasis. Bull. Cancer.

[2] Pachmayr E., Treese C., Stein U. (2017). Underlying Mechanisms for Distant Metastasis—Molecular Biology. Visc. Med..

[3] Zhu T., Bao X., Chen M., Lin R., Zhuyan J., Zhen T., Xing K., Zhou W., Zhu S. (2020). Mechanisms and Future of Non-Small Cell Lung Cancer Metastasis. Front. Oncol..

[4] Hudeckova M., Koucky V., Rottenberg J., Gal B. (2021). Gene Mutations in Circulating Tumour DNA as a Diagnostic and Prognostic Marker in Head and Neck Cancer-A Systematic Review. Biomedicines.

[5] Fan Y., Wang Y., Fu S., Yang L., Lin S., Fan Q., Wen Q. (2018). The diagnostic role of DNA methylation in sporadic endometrial cancer: A systematic review and meta-analysis. Oncotarget.

[6] Ogden G.R., Macluskey M. (2000). An overview of the prevention of oral cancer and diagnostic markers of malignant change: 1. Prevention. Dent. Update.

[7] Raja V., Farajzadegan Z., Mansourian M., Ghasemi K., Aboutalebi M.S., Nouri R., Mokarian F. (2022). Diagnostic Value of Nonacid Nucleic Blood Tumor Marker Panels in Early Diagnosing Breast Cancer: A Systematic Review and Network Meta-Analysis. Dis. Markers.

[8] Hou X., Yang L., Wang K., Zhou Y., Li Q., Kong F., Liu X., He J. (2021). HELLS, a chromatin remodeler is highly expressed in pancreatic cancer and downregulation of it impairs tumor growth and sensitizes to cisplatin by reexpressing the tumor suppressor TGFBR3. Cancer Med..

[9] Kollarovic G., Topping C.E., Shaw E.P., Chambers A.L. (2020). The human HELLS chromatin remodelling protein promotes end resection to facilitate homologous recombination and contributes to DSB repair within heterochromatin. Nucleic Acids Res..

[10] Robinson M.H., Maximov V., Lallani S., Farooq H., Taylor M.D., Read R.D., Kenney A.M. (2019). Upregulation of the chromatin remodeler HELLS is mediated by YAP1 in Sonic Hedgehog Medulloblastoma. Sci. Rep..

[11] Zhang G., Dong Z., Prager B.C., Kim L.J., Wu Q., Gimple R.C., Wang X., Bao S., Hamerlik P., Rich J.N. (2019). Chromatin remodeler HELLS maintains glioma stem cells through E2F3 and MYC. JCI Insight.

[12] Chi C., Liu N., Yue L., Qi W.W., Xu L.L., Qiu W.S. (2015). RTN4/Nogo is an independent prognostic marker for gastric cancer: Preliminary results. Eur. Rev. Med. Pharmacol. Sci..

[13] Guo Z., Zhang X., Zhu H., Zhong N., Luo X., Zhang Y., Tu F., Zhong J., Wang X., He J. (2021). TELO2 induced progression of colorectal cancer by binding with RICTOR through mTORC2. Oncol. Rep..

[14] Wang F., Lou X., Zou Y., Hu D., Liu J., Ning J., Jiao Y., Zhang Z., Yang F., Fan L. (2020). Overexpression of Rictor protein and Rictor-H. pylori interaction has impact on tumor progression and prognosis in patients with gastric cancer. Folia Histochem. Cytobiol..

[15] Parte S.C., Batra S.K., Kakar S.S. (2018). Characterization of stem cell and cancer stem cell populations in ovary and ovarian tumors. J. Ovarian Res..

[16] Noto Z., Yoshida T., Okabe M., Koike C., Fathy M., Tsuno H., Tomihara K., Arai N., Noguchi M., Nikaido T. (2013). CD44 and SSEA-4 positive cells in an oral cancer cell line HSC-4 possess cancer stem-like cell characteristics. Oral Oncol..

[17] Yang L., Yang J., Jacobson B., Gilbertsen A., Smith K., Higgins L., Guerrero C., Xia H., Henke C.A., Lin J. (2022). SFPQ Promotes Lung Cancer Malignancy via Regulation of CD44 v6 Expression. Front. Oncol..

[18] Ji Q., Zhang L., Liu X., Zhou L., Wang W., Han Z., Sui H., Tang Y., Wang Y., Liu N. (2014). Long non-coding RNA MALAT1 promotes tumour growth and metastasis in colorectal cancer through binding to SFPQ and releasing oncogene PTBP2 from SFPQ/PTBP2 complex. Br. J. Cancer.

[19] Katano-Toki A., Yoshino S., Nakajima Y., Tomaru T., Nishikido A., Ishida E., Horiguchi K., Saito T., Ozawa A., Satoh T. (2021). SFPQ associated with a co-activator for PPARgamma, HELZ2, regulates key nuclear factors for adipocyte differentiation. Biochem. Biophys. Res. Commun..

[20] Klotz-Noack K., Klinger B., Rivera M., Bublitz N., Uhlitz F., Riemer P., Luthen M., Sell T., Kasack K., Gastl B. (2020). SFPQ Depletion Is Synthetically Lethal with BRAF(V600E) in Colorectal Cancer Cells. Cell Rep..

[21] Lu D.Y., Mao X.H., Zhou Y.H., Yan X.L., Wang W.P., Zheng Y.B., Xiao J.J., Zhang P., Wang J.G., Ashwani N. (2014). RTN4 3′-UTR insertion/deletion polymorphism and susceptibility to non-small cell lung cancer in Chinese Han population. Asian Pac. J. Cancer Prev..

[22] Cheng H., Zou Y., Ross J.S., Wang K., Liu X., Halmos B., Ali S.M., Liu H., Verma A., Montagna C. (2015). RICTOR Amplification Defines a Novel Subset of Patients with Lung Cancer Who May Benefit from Treatment with mTORC1/2 Inhibitors. Cancer Discov..

[23] Sakre N., Wildey G., Behtaj M., Kresak A., Yang M., Fu P., Dowlati A. (2017). RICTOR amplification identifies a subgroup in small cell lung cancer and predicts response to drugs targeting mTOR. Oncotarget.

[24] Schmidt K.M., Hellerbrand C., Ruemmele P., Michalski C.W., Kong B., Kroemer A., Hackl C., Schlitt H.J., Geissler E.K., Lang S.A. (2017). Inhibition of mTORC2 component RICTOR impairs tumor growth in pancreatic cancer models. Oncotarget.

[25] Wen F.F., Li X.Y., Li Y.Y., He S., Xu X.Y., Liu Y.H., Liu L., Wu S.H. (2020). Expression of Raptor and Rictor and their relationships with angiogenesis in colorectal cancer. Neoplasma.

[26] Wong C.K., Lambert A.W., Ozturk S., Papageorgis P., Lopez D., Shen N., Sen Z., Abdolmaleky H.M., Gyorffy B., Feng H. (2020). Targeting RICTOR Sensitizes SMAD4-Negative Colon Cancer to Irinotecan. Mol. Cancer Res..

[27] Chen L., Su Y., Yin B., Li S., Cheng X., He Y., Jia C. (2022). LARP6 Regulates Keloid Fibroblast Proliferation, Invasion, and Ability to Synthesize Collagen. J. Investig. Dermatol..

[28] Sheel A., Shao R., Brown C., Johnson J., Hamilton A., Sun D., Oppenheimer J., Smith W., Visconti P.E., Markstein M. (2020). Acheron/Larp6 Is a Survival Protein That Protects Skeletal Muscle From Programmed Cell Death During Development. Front. Cell Dev. Biol..

[29] Stefanovic B., Manojlovic Z., Vied C., Badger C.D., Stefanovic L. (2019). Discovery and evaluation of inhibitor of LARP6 as specific antifibrotic compound. Sci. Rep..

[30] Zhang Y., Stefanovic B. (2017). mTORC1 phosphorylates LARP6 to stimulate type I collagen expression. Sci. Rep..

[31] Liu X., Hou X., Zhou Y., Li Q., Kong F., Yan S., Lei S., Xiong L., He J. (2019). Downregulation of the Helicase Lymphoid-Specific (HELLS) Gene Impairs Cell Proliferation and Induces Cell Cycle Arrest in Colorectal Cancer Cells. OncoTargets Ther..

[32] Waseem A., Ali M., Odell E.W., Fortune F., Teh M.T. (2010). Downstream targets of FOXM1: CEP55 and HELLS are cancer progression markers of head and neck squamous cell carcinoma. Oral Oncol..

[33] Lim Y.W., James D., Huang J., Lee M. (2020). The Emerging Role of the RNA-Binding Protein SFPQ in Neuronal Function and Neurodegeneration. Int. J. Mol. Sci..

[34] Grasso D., Bintz J., Lomberk G., Molejon M.I., Loncle C., Garcia M.N., Lopez M.B., Urrutia R., Iovanna J.L. (2015). Pivotal Role of the Chromatin Protein Nupr1 in Kras-Induced Senescence and Transformation. Sci. Rep..

[35] Kanazawa T., Misawa K., Misawa Y., Uehara T., Fukushima H., Kusaka G., Maruta M., Carey T.E. (2015). G-Protein-Coupled Receptors: Next Generation Therapeutic Targets in Head and Neck Cancer?. Toxins.

[36] Kim K., Doi A., Wen B., Ng K., Zhao R., Cahan P., Kim J., Aryee M.J., Ji H., Ehrlich L.I. (2010). Epigenetic memory in induced pluripotent stem cells. Nature.

[37] Draht M.X.G., Goudkade D., Koch A., Grabsch H.I., Weijenberg M.P., van Engeland M., Melotte V., Smits K.M. (2018). Prognostic DNA methylation markers for sporadic colorectal cancer: A systematic review. Clin. Epigenetics.

[38] Gurung P.M.S., Barnett A.R., Wilson J.S., Hudson J., Ward D.G., Messing E.M., Bryan R.T. (2020). Prognostic DNA Methylation Biomarkers in High-risk Non-muscle-invasive Bladder Cancer: A Systematic Review to Identify Loci for Prospective Validation. Eur. Urol. Focus.

[39] Nowacka-Zawisza M., Wisnik E. (2017). DNA methylation and histone modifications as epigenetic regulation in prostate cancer (Review). Oncol. Rep..

[40] Ahmed Aglan S., Mohamad Zaki A., Sobhy El Sedfy A., Gaber El-Sheredy H., Hussein Elgaddar O. (2022). O6-Methylguanine-DNA Methyltransferase and ATP-Binding Cassette Membrane Transporter G2 Promotor Methylation: Can Predict the Response to Chemotherapy in Advanced Breast Cancer?. Rep. Biochem. Mol. Biol..

[41] Michalowska-Sawczyn M., Grzywacz A., Masiak J., Chmielowiec K., Chmielowiec J., Chycki J., Maculewicz E., Cieszczyk P. (2021). Associations Between Physical Effort and DNA Methylation in the Promotor Region of the Dopamine Transporter Gene (DAT1). J. Hum. Kinet..

[42] Yildiz O.G., Aslan D., Akalin H., Erdem Y., Canoz O., Aytekin A., Ozoner S., Dundar M. (2020). The Effects of O(6)-methyl Guanine DNA-methyl Transferase Promotor Methylation and CpG1, CpG2, CpG3 and CpG4 Methylation on Treatment Response and their Prognostic Significance in Patients with Glioblastoma. Balk. J. Med. Genet..

[43] de Jong A., Pietersma H., Cordes M., Kuipers O.P., Kok J. (2012). PePPER: A webserver for prediction of prokaryote promoter elements and regulons. BMC Genom..

[44] Satterstrom F.K., Haigis M.C. (2014). Luciferase-based reporter to monitor the transcriptional activity of the SIRT3 promoter. Methods Enzymol..

[45] Qi W., Li X., Kang J. (2014). Advances in the study of serum tumor markers of lung cancer. J. Cancer Res. Ther..

[46] Molina R., Marrades R.M., Auge J.M., Escudero J.M., Vinolas N., Reguart N., Ramirez J., Filella X., Molins L., Agusti A. (2016). Assessment of a Combined Panel of Six Serum Tumor Markers for Lung Cancer. Am. J. Respir. Crit. Care Med..

[47] Zhang X., Hu M., Lyu X., Li C., Thannickal V.J., Sanders Y.Y. (2017). DNA methylation regulated gene expression in organ fibrosis. Biochim. Biophys. Acta Mol. Basis Dis..

[48] Su J., Shao X., Liu H., Liu S., Wu Q., Zhang Y. (2012). Genome-wide dynamic changes of DNA methylation of repetitive elements in human embryonic stem cells and fetal fibroblasts. Genomics.

[49] Bar-Nur O., Russ H.A., Efrat S., Benvenisty N. (2011). Epigenetic memory and preferential lineage-specific differentiation in induced pluripotent stem cells derived from human pancreatic islet beta cells. Cell Stem Cell.

[50] Calvanese V., Horrillo A., Hmadcha A., Suarez-Alvarez B., Fernandez A.F., Lara E., Casado S., Menendez P., Bueno C., Garcia-Castro J. (2008). Cancer genes hypermethylated in human embryonic stem cells. PLoS ONE.

[51] Rayner S.L., Cheng F., Hogan A.L., Grima N., Yang S., Ke Y.D., Au C.G., Morsch M., De Luca A., Davidson J.M. (2021). ALS/FTD-causing mutation in cyclin F causes the dysregulation of SFPQ. Hum. Mol. Genet..

[52] Taylor R., Hamid F., Fielding T., Gordon P.M., Maloney M., Makeyev E.V., Houart C. (2022). Prematurely terminated intron-retaining mRNAs invade axons in SFPQ null-driven neurodegeneration and are a hallmark of ALS. Nat. Commun..

[53] Widagdo J., Udagedara S., Bhembre N., Tan J.Z.A., Neureiter L., Huang J., Anggono V., Lee M. (2022). Familial ALS-associated SFPQ variants promote the formation of SFPQ cytoplasmic aggregates in primary neurons. Open Biol..

[54] Zhang D.G., Jiang A.G., Lu H.Y., Zhang L.X., Gao X.Y. (2015). Isolation, cultivation and identification of human lung adenocarcinoma stem cells. Oncol. Lett..

[55] Yang L., Xia H., Smith K., Gilbertsen A., Beisang D., Kuo J., Bitterman P.B., Henke C.A. (2021). A CD44/Brg1 nuclear complex confers mesenchymal progenitor cells with enhanced fibrogenicity in idiopathic pulmonary fibrosis. JCI Insight.

[56] Zheng C., Sun Y.H., Ye X.L., Chen H.Q., Ji H.B. (2011). Establishment and characterization of primary lung cancer cell lines from Chinese population. Acta Pharmacol. Sin..

[57] Kramer A., Green J., Pollard J., Tugendreich S. (2014). Causal analysis approaches in Ingenuity Pathway Analysis. Bioinformatics.

[58] Yu J., Gu X., Yi S. (2016). Ingenuity Pathway Analysis of Gene Expression Profiles in Distal Nerve Stump following Nerve Injury: Insights into Wallerian Degeneration. Front. Cell. Neurosci..

[59] Bashtrykov P., Jeltsch A. (2018). DNA Methylation Analysis by Bisulfite Conversion Coupled to Double Multiplexed Amplicon-Based Next-Generation Sequencing (NGS). Methods Mol. Biol..

[60] Claus R., Lucas D.M., Stilgenbauer S., Ruppert A.S., Yu L., Zucknick M., Mertens D., Buhler A., Oakes C.C., Larson R.A. (2012). Quantitative DNA methylation analysis identifies a single CpG dinucleotide important for ZAP-70 expression and predictive of prognosis in chronic lymphocytic leukemia. J. Clin. Oncol..

